# Are single nucleotide polymorphisms rs7903146 and rs12255372 in transcription factor 7-like 2 gene associated with an increased risk for gestational diabetes mellitus in Egyptian women?

**DOI:** 10.1186/s43141-021-00272-6

**Published:** 2021-11-01

**Authors:** Taghreed A. Shalabi, Khalda S. Amr, Mai M. Shaker

**Affiliations:** 1 Prenatal Diagnosis and Fetal Medicine Department, Human Genetics and Genome Research Institute, National Research Centre, Cairo, Egypt; 2 Medical Molecular Genetics Department, Human Genetics and Genome Research Institute, National Research Centre, Cairo, Egypt

**Keywords:** Gestational diabetes, Pregnancy, *TCF7L2*, Variants, *rs7903146*, *rs12255372*

## Abstract

**Background:**

Genetic variants in the transcription factor 7-like 2 (*TCF7L2*) gene are related with type 2 diabetes (T2D) and gestational diabetes mellitus (GDM) in various populations, but there are not enough statistics regarding GDM among Egyptian women. We aimed by this study to evaluate the effect of two polymorphisms of *rs7903146* and *rs12255372* in the *TCF7L2* gene with the development of GDM among Egyptian women.

**Results:**

We enrolled 114 pregnant women with normal glucose tolerance and 114 with GDM according to the International Association of Diabetes and Pregnancy Study Groups (IADPSG) guidelines. We gathered records on blood pressure, body mass index (BMI), blood glucose level, hemoglobin A1C (HbA1c), and lipid profile. The genotyping of *rs7903146* and *rs12255372* polymorphisms was carried out using polymerase chain reaction–restriction fragment length polymorphism (PCR-RFLP). The statistical significance of prepregnancy BMI, fasting blood sugar (FBS), HbA1c, low-density lipoprotein (LDL), and total cholesterol (Tch) was higher, *P* < 0.001, in GDM women in comparison to pregnant women without GDM. CT and TT genotypes in rs7903146 SNP were 46.5% vs. 54%, *P* <0.04, *OR*; *CI* = 1.9 (1.0 to 3.78); TT carriers were 37.7% vs. 9.6%, *P* <0.001, *OR* (*CI*) = 8.9 (3.7–21.1), respectively. For the *TCFL2* gene rs12255372 SNP, GT carriers were 48.2% vs. 39.5%, *P*= 0.004, *OR* (*CI*) = 2.3 (1.3–4.2), while TT carriers were 24.6% vs. 7.9%, *P* < 0.001, *OR* (*CI*) = 6 (2.5–14.3).

**Conclusion:**

The study showed there is a significantly higher incidence of CT/TT genotypes in *rs7903146* SNP and GT/TT genotypes in *rs12255372* SNP in *TCF7L2* gene among GDM women in comparison to healthy pregnant women (controls).

## Background

Gestational diabetes mellitus (GDM) is a kind of diabetes that is acquired during the gestation period. It is important to distinguish it from overt diabetes. There is a continuous increase in the incidence of GDM [1]. In 2020, the Center of Disease Control (CDC) reported that the prevalence of GDM is around 10% [2]. However, incidence differs from one population to another. There are only 2 existing studies regarding the prevalence of GDM among Egyptians. One took place in Upper Egypt where incidence was about 17.5% and the other in the Delta region (Menoufia governorate) with an incidence of 6%. Among the American population, the occurrence of GDM is around 7% [3, 4]. In Gulf countries, the occurrence of GDM varies between 4.2 and 24.9% [5]. In 2010, many countries followed the guidelines of the International screening of Gestational Diabetes according to the Association of Diabetes and Pregnancy Study Groups (IADPSG) [6]. A couple of years later, after following IADPSG guidelines, the incidence of GDM has been assessed to be approximately 17% worldwide and in specific 10% among North Americans and 25% in Southeast Asia [7].

Due to the global widespread of obesity among young fertile women, the prevalence of GDM is in a constant rapid increase [8]. Hyperglycemic state during the gestation period is directly correlated with the increase in the number of feto-maternal co-morbidities [9]. Type 2 diabetes (T2D) is more common to evolve among pregnant women with a history of GDM as well as their offsprings are highly vulnerable to having diabetes and obesity during their grownup life [10]. Obesity and family history for diabetes both are important risk factors associated with GDM and T2D [11].

Due to the resemblance between GDM and T2D, it was documented that both might have some genetic factors in common such as single nucleotide polymorphisms (SNPs) which increase the risk for diabetes [12]. *TCF7L2* is positioned at chromosome 10q25.3 and its amino acid controls hyperglycemic state in the blood [13]. The actual action of *TCF7L2* in developing DM is still under debate though there are theories that DM might occur due to malfunction or decreased number in pancreatic islets [14]. It is assumed that the *TCF7L2* gene controls proglucagon expression inside entero-endocrine cells [15]. *TCF7L2* is important in the propagation of pancreatic B-cell as well as the production of several incretin hormones, glucose-dependent insulin-tropic peptide (GLP-1) which are released from entero-endocrine cells. GLP-1 also stimulates insulin secretion [16]. *TCF7L2* regulates the metabolism of hepatic glucose mainly through inhibiting gluconeogenesis [17, 18]. *TCF7L2* gene has been related to T2D in various populations [16, 19, 20]. Intronic regions of the *TCF7L2* gene have variants which are well known as strong risk genetic markers for T2D [21]. *TCF7L2* polymorphisms have a dual effect on blood glucose level and insulin excretion [16]. *TCF7L2* polymorphism *rs7903146* has been related with GDM in Danish [22], Australian [20], Greek [23], and Swedish populations [19] as well as other populations [19, 24, 25]. The aim of this study is to evaluate the association of two polymorphisms of *rs7903146* and *rs12255372* in the TCF7L2 gene with the risk of development of GDM among Egyptian women.

## Methods

A total of 228 Egyptian women were examined. Healthy pregnant women were classified as the control group (*n* = 114) and pregnant women with GDM as the GDM group (*n* = 114). Screening for GDM using oral glucose tolerance test (OGTT) was done for the participants according to the IADPSG guidelines [26]. Participants were given 75 g of glucose. Fasting and 2-h blood glucose levels were assessed after that. The diagnosis of GDM was made after which any one of the following plasma glucose values were exceeded: if the fasting plasma glucose was 92 mg/dL or more or when the 2-h value is 153 mg/dL or more. Values (fasting ≥ 126 mg/dL or 2 h postprandial ≥ 200 mg/dL) specify overt diabetes mellitus (DM), which was excluded from this study. The study was conducted from the period of 2019–2021. Participants were referred to our prenatal diagnosis and fetal medicine department. The study was done after approval was obtained from the ethical committee (Medical research ethical Committee). With approval reference number 19266, it conforms to the provisions of the Declaration of Helsinki. Informed written consent was obtained from all participants in the study. The control group was selected from low-risk pregnant women who came for regular follow-up at our clinic unit to do a 20-week ultrasound. Women who were appropriate to be in the control group in the study were called at 28 weeks to check a normal blood glucose level then accordingly were involved in the study protocol.

### Inclusion criteria

All non-diabetic pregnant women, coming for antenatal care, were assessed. Fasting and 2h postprandial, HbA1c were done at 12 weeks for high-risk pregnancies for GDM (first degree relative with diabetes, history of GDM during previous pregnancies, history of delivering overweight babies > 4 kg or prepregnancy overweight; BMI > 25kg/m^2^, age 20–45 years, and fetal Doppler scan within the normal reference range) [27]. Then, reassessment for the high-risk group was taken at 24–28 weeks, and whoever tested positive for hyperglycemias was included in the case group.

### Exclusion criteria

Pregnant women with diabetes history before pregnancy or overt diabetes mellitus (DM) (fasting ≥ 126 mg/dL or 2 h postprandial ≥ 200mg/dL) were excluded from the study. Women known to be hypertensive were also excluded from the study. Any woman who is a smoker or having any pathology other than GDM before or during pregnancy was excluded.

### Clinical and laboratory data

Clinical and anthropometric records were collected from patient files containing fasting blood glucose, postprandial, and HA1c, and fat metabolism was evaluated (total cholesterol (TCh), low-density lipoprotein (LDL), high-density lipoprotein cholesterol (HDL), serum triglycerides (TG)).

### Genotyping

**Fig. 1:**
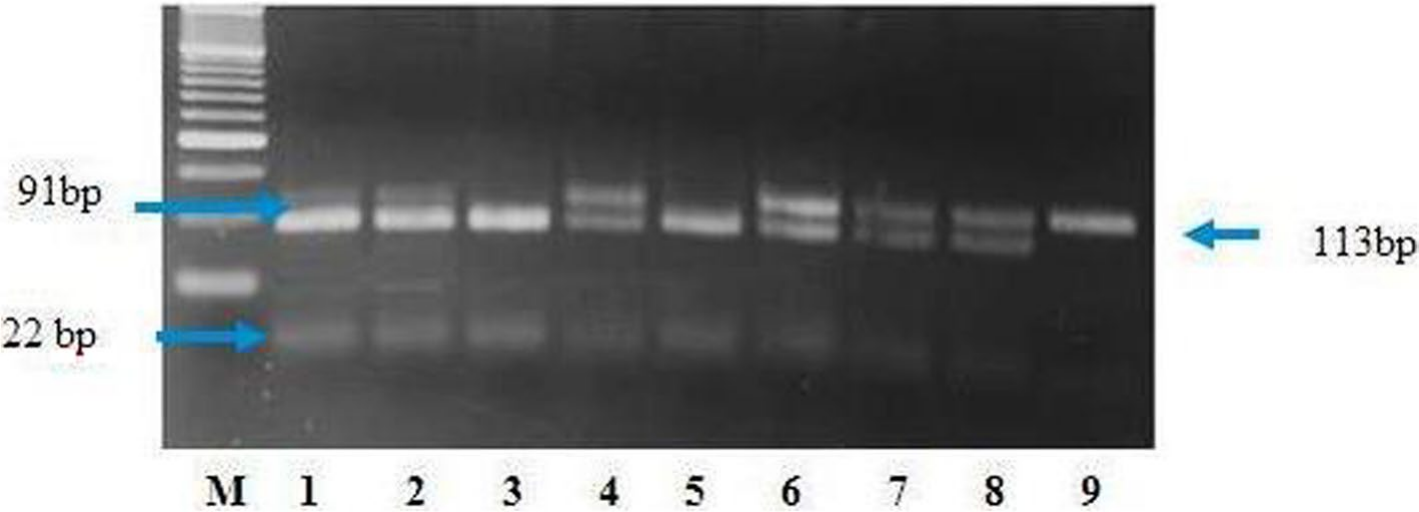
A 3.5% agarose gel illustrating digestion of the PCR products with RsaI for detection of the *rs7903146 polymorphism*. Lanes 1–3 and 5: 4 samples with the C/C genotype (91 and 22 bp). Lanes 4, 6, 7, and 8: 4 samples with the T/C genotype (113, 91, and 22 bp). Lane 9: one sample with the T/T genotype (113 bp). Lane M: 50-bp ladder (Fermentas, Germany)

DNA was extracted from whole blood using the “salting out” method [28] and normalized to 20 ng/μL for the following analyses. Only samples with 280/260 absorbance ratios of 1.8 to 2.0 (NanoDrop, Thermo Scientific) were used in this study. *TCF7L2* gene polymorphisms of *rs12255372* and *rs7903146* gene were carried out using PCR-RFLP analysis as described by Bodhini et al. [29] and Szepietowska et al. [30]. Two sets of primers were used to amplify the loci in the *TCF7L2* gene containing the two polymorphisms. The primers used and the size of the PCR products and restriction enzyme used are listed in Table 1 [29]. PCR was carried out in a 50-μl total final volume containing 200 μM dNTPs (Finzyme, Finland), 10 pmole of each primer, 2U of Taq polymerase (Finzyme, Finland), and 500 ng DNA. Thermal cycling conditions were as follows: denaturation at 95°C for 10 min, followed by 30 cycles of denaturation at 95 °C for 50 s, annealing at 56 °C 50 s, and elongation at 72 °C for 50 s followed by a final elongation of 5 min. Ten microliters of a successfully amplified PCR fragment containing the rs7903146 polymorphism was digested with RsaI enzyme (Fermentas, Germany) and incubated at 37 °C for 3 h and the fragments were run on 3.5% agarose gel stained with ethidium bromide and analyzed under ultraviolet light. The RsaI enzyme cuts digested PCR product (113 bp) into two fragments (91 and 22 bp ) in the presence of the C allele as shown in Fig. 1.

**Table 1:** Sequence of primers, size of the PCR products, and restriction enzyme used in genotyping of the *rs7903146* and *rs12255372* in the *TCF7L2* gene

Polymorphism	Sequence of primers	Size of PCR product (bp)	Restriction enzyme
*rs7903146* (C/T)	Forward: 5′-CTGGAAACTAAGGCGTGA GG-3′ Reverse: 5′-GGGTCGATGTTGTTGAGC TT-3′	113	RsaI
*rs12255372* (G/T)	Forward: 5′-GAGAGCTAAGCA CTT TTTAGG TA-3′ Reverse: 5′-CCTCATACGGCAATTAAATTATACA-3′	346	Tsp509I

**Fig. 2:**
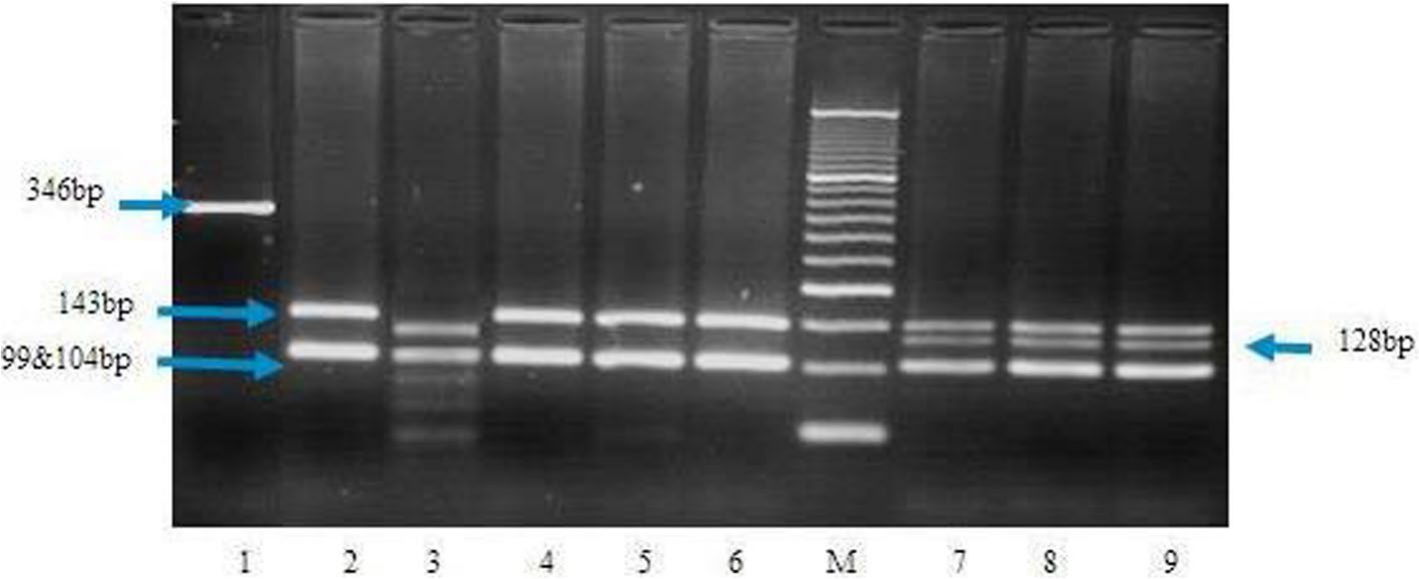
A 3.5% agarose gel illustrating digestion of the PCR products with Tsp509I for detection of the *rs12255372 polymorphism.* Lane 1: undigested PCR product (346 bp). Lanes 2 and 4–6: 4 patients with the G/G genotype (143, 104, 99 bp). Lane 3: 1 patient with the T/T genotype (128, 104, 99, 17 bp). Lanes 7–9: three patients with the G/T genotype (143, 128, 104, 99, 17 bp). Lane M: size marker (50-bp DNA ladder, Fermentas, Germany)

On the other hand, ten microliters of PCR fragments containing the rs12255372 was digested with Tsp509I (Fermentas, Germany) and incubated at 65 °C for 3 h and the fragments were run in 3.5% agarose gel stained with ethidium bromide and analyzed under ultraviolet light. The Tsp509I enzyme cuts PCR product (346 bp) in three fragments (143, 104, 99 bp) in the presence of the G allele while the T allele creates an additional site for the enzyme resulting in five fragments (128, 104, 104, 99, 17 bp) as shown in Fig. 2.

### Statistical analysis

Statistical analysis was done using IBM© SPSS© Statistics version 22 (IBM© Corp., Armonk, NY, USA). Normality was tested using the Kolmogorov–Smirnov test. Comparisons of parameters with normal distribution were performed using the Student *t*-test for independent samples. Categorical variables were compared using the Fisher exact test (two-tailed) or the chi-square test, as appropriate. Allele frequencies and Hardy–Weinberg (HW) equilibrium were verified using the chi-square test. Probability lower than 5% (*P* < 0.05) was considered significant. The genotypic relative risk was assessed by comparing the GDM with the control group and calculating the odds ratio and 95% confidence interval.

## Results

The anthropometric characteristics and laboratory data of all participants are demonstrated in Table 2.

**Table 2:** Anthropometric and lab parameters among studied groups

	GDM (***n***=114)	Control (***n***=114)	***P*** value
**Maternal age (years)**	27.9 ± 3.8	27.2 ± 3.2	0.05
**BMI (kg/m**^**2**^**)**	28 ± 2	26.3 ± 4.5	< 0.001
**SPB (mmHg)**	127± 3	120± 6	< 0.001
**DBP (mmHg)**	77 ± 4	73 ± 5	< 0.001
**HA1c % (1st visit)**	5.3	4.9 ± 0.3	< 0.001
**FBS (mg/dL)**	118 ± 5.5	80 ± 9	< 0.001
**2h postprandial**	179 ± 8.7	114.5 ± 17.9	< 0.001
**Creatinine (mg/dL)**	0.9 ± 0.2	1.0 ± 0.7	0.45
**Urea (mg/dL)**	12.6 ± 3.5	11.8 ± 3.1	0.79
**LDL (mg/dL)**	101 ± 4.5	100 ± 4.6	0.02
**HDL (mg/dL)**	56.1 ± 3.4	55.8 ± 3.2	0.28
**Cholesterol (mg/dL)**	153 ± 5.3	149 ± 6	< 0.001
**Triglycerides (mg/dL)**	123 ± 5.5	121 ± 5.6	0.07
**History of babies > 4kg**	8/114	7/114	0.78
**Family history DM**	65/114	45/11	0.01

*BMI* body mass index, *HA1c* hemoglobin A1c, *FBS* fasting blood sugar, *LDL* low-density lipoprotein, *HDL* high-density lipoprotein, *GDM* gestational diabetes mellitus, *SBP* systolic blood pressure, *DBP* diastolic blood pressure, *DM* diabetes mellitus

*P* < 0.05 is considered significant

Both groups were comparable in age, *P*= 0.05. GDM patients were significantly weightier with a higher body mass index, *P*= 0.05. Blood pressure was within the normal range, *P* <0.001, but still significantly higher among the GDM group in comparison to healthy pregnant women in the control group. Family history of diabetes was more prominent among the GDM group, *P*=0.01. Among the GDM group, fasting glucose and glycated hemoglobin were lying in the normal reference range, signifying that those patients had a past good glycemic index. Postprandial hyperglycemia was observed among the GDM group, which was more than 153 mg/dL but less than 200 mg/dL indicating the presence of GDM and the absence of preexisting overt diabetes according to IADPSG guidelines [26]. Those results display the occurrence of a hyperglycemic surge in the GDM group, which is well-matched with the studied disease as shown in Table 1. Lipid profile was normal apart from total cholesterol (Tch) and LDL which were significantly higher among the GDM group in comparison to controls, *P* = 0.02 and 0.001, respectively.

All genotypes in both groups were in consistency with HW equilibrium (*P N* 0.05). Regarding *rs7903146* SNP, CT and TT genotypes were more prominent among GDM in comparison to the control group; CT carriers among the GDM group were 46.5% vs. 54% and TT carriers were 37.7% vs. 9.6%, respectively. Consequently, dominant and recessive models were highly significant among the GDM group in comparison to the control group. As for the *rs12255372* SNP, GT and TT genotypes were also more prominent among the GDM group in comparison to the control group; GT carriers among GDM were 48.2% vs. 39.5%. TT carriers were 24.6% vs. 7.9%. Consequently, dominant and recessive models were highly significant among the GDM group in comparison to the control group. The T allele in *rs7903146* is 61% among the GDM group vs. 36.8% among the control group, having a *P* value of < 0.001, *OR* 2.6, and *CI* 1.8–3.9. The T allele in *rs12255372* SNP among the GDM group was 51.3% vs. 75.5% among the control group having a *P* value of < 0.001, *OR* 2.9, and *CI* 1.9–4.4 as shown in Table 3.

**Table 3:** *TCF7L2* genotyping and allele frequencies for GDM cases and controls

	GDM *N* = 114 (%)	Controls *N* = 114 (%)	***P*** value; ***OR*** (***95th CI***)
***rs7903146*** **(C/T)**			
CC	18 (15.2%)	41 (35%)	***Ref***
CT	53 (46.5%)	62 (54%)	0.04; 1.9 (1.0 to 3.78)
TT	43 (37.7%)	11 (9.6%)	<0.001; 8.9 (3.7–21.1)
CT+TT vs. CC	96 (84.2%)	73 (64%)	<0.001; 3 (1.5–5.6)
TT vs. CC +CT	43 (47.7%)	11 (9.6%)	<0.001; 5.6 (2.7–11.7)
HWE	0.8	0.07	
*rs7903146* (C/T)			
C	39%	63.1%	***Ref***
T	61%	36.8%	<0.001; 2.6 (1.8–3.9)
***rs12255372*** **(G/T)**			
GG	31 (27.2%)	60 (52.6%)	***Ref***
GT	55 (48.2 %)	45 (39.5%)	0.004; 2.3 (1.3–4.2 )
TT	28 (24.6%)	9 (7.9%)	<0.001; 6 (2.5–14.3)
GT+TT vs. GG	83 (72.8%)	54 (47.7%)	<0.001; 2.9 (1.7–5.1)
TT vs. GG +GT	28 (24.6%)	9 (9.7%)	0.001; 3.7 (1.7–8.4 )
HWE	0.7	0.8	
*rs12255372* (G/T)			
G	48.7%	24.3%	***Ref***
T	51.3%	75.5%	<0.001; 2.9 (1.9–4.4 )

*OR* odds ratio, *CI* confidence interval, *HWE* Hardy–Weinberg equation

*P*<0.05 is considered significant

## Discussion

GDM is broadly studied, yet its pathophysiology is not clearly understood. In the meantime, it is important to consider women either with a positive family history of T2D or had a previous history of GDM are at a high risk for developing GDM in upcoming future pregnancies. It is hypothesized that GDM might have similar risk factors and genetic liabilities with T2D [31]. The approximate incidence of acquiring diabetes is nearly 60% in the upcoming future among females with GDM. Associated factors predicting the development of T2D afterwards stay comparable between several cohorts [32]. We aimed by this study to evaluate the effect of two polymorphisms of *rs7903146* and *rs12255372* in the *TCF7L2* gene with the development of GDM among Egyptian women.

Among different populations, *TCF7L2* is known to be the most common risk gene for T2D. It is the most frequently examined gene among women with GDM [33, 34]. The *TCF7L2* gene polymorphisms were documented to be correlated with GDM in many populations [24, 35, 36]. Conferring to the literature, there is not a highlight on the specific geographical area for *TCF7L2* SNP allele frequency [37]. In our study, clinical parameters including BMI and hypertension measurements among the GDM group were comparable with other published studies that stated that pregnant women with GDM have a higher BMI and more frequency of hypertension in comparison to healthy controls [38, 39]. Throughout the normal gestation period, Tch and TG levels in the maternal blood are more prone to a sustained increase [40]. Those fluctuations in lipid profile are intensified with the existence of GDM [41]. This explains the difference found in the lipid profiles among the studied groups as shown in Table 1. Percentages of the other laboratory parameters were as predicted, and the results were comparable to other reported studies representing GDM patients with acceptable glycemic index as shown in Table 1 [42].

Women enrolled in this study were entirely Egyptians. CT and TT genotypes in rs7903146 SNP were more prominent among the GDM group in comparison to the control group. Women carrying the T allele have a nearly 2-fold increase risk for GDM. Regarding *rs12255372* SNP, GT and TT genotypes were more prominent among the GDM group in comparison to the control group. Women carrying the T allele have a nearly 3-fold increase risk for GDM.

Reyes-López et al. reported that poor β-cell activity, maternal prepregnancy overweight, and *rs12255372* risk allele are all independent risk factors related with the evolving of gestational diabetes [43]. Dan Ye and his colleagues studied 18 SNPs within *TCF7L2* and found out that *rs290487*, *rs6585194*, and *rs7094463* polymorphisms are significantly related with the existence of GDM [31]. A meta-analysis study done by Chang and his colleagues reported a direct relationship between *TCF7L2* alleles and the presence of chronic pregnancy hyperglycemia [44]. The study reported that six polymorphisms in the *TCF7L2* gene had a highly significant correlation with the increased risk of GDM among the entire population. Chang and his colleagues also highlighted the high significance found between diverse ethnic populations [44]. Reyes-Lopez and his colleagues reported that *rs12255372* is more prominent among women increasing the liability for insulin glucose tolerance (IGT) status as well as increasing the frequency for developing T2D [42]. Potasso and his colleagues reported that T mutant allele in *TCF7L2 rs7903146* is related to the loss of initial postprandial glycemic control and stressed on the necessity of insulin treatment among pregnant women with chronic hyperglycemia, even if other risk interfering factors like BMI had been regulated [41]. There is an increased possibility of having T2D in different cohorts especially among CT/TT genotype carriers of SNP *rs7903146* [15]. In a long time study of around 22 years of follow-up, mutant T allele carriers had a poor insulin feedback mechanism to random OGTT as well as greater chance for developing T2D in comparison to CC genotype carriers [13]. Lin and his colleagues conducted a meta-analysis and they reported a significant correlation between mutant allele of rs7903146 with the high chances of acquiring GDM among all genomic models in the entire population of white and Hispanic/Latino as well as Asian subcategories. Lin and his colleagues also stated that homozygous TT allele carriers for the SNP *rs7903146* among Asians were highly correlated with the risk of GDM (*OR* = 2.08) followed by Hispanics/Latinos (*OR* = 1.80) and whites (*OR* = 1.51) [45]. It is reported that *TCF7L2* has a strong impact on the breakdown and absorption of glucose and lipids; therefore, it is hypothesized that TT carriers might have a greater chance for developing cardiovascular diseases [40]. Francaite et al. reported that CT/TT genotypes in rs7903146 SNP and GT/TT genotypes in *rs12255372* SNP in *TCF7L2* gene were more prominent among GDM Lithuanian women in comparison to women from the general population [46]. In contrast to our results, de Melo et al. reported that *rs12255372* and *rs7903146* were not associated with GDM in the Euro-Brazilian population [47]. Siudak et al. also found no relationship between *rs7903146* polymorphism of the *TCF7L2* gene and GDM in the Polish population [48]. A study was conducted in North India on 115 women with GDM, and *TCF7L2* variants *rs 7903146* and *rs 12255372* showed no association with GDM among the Indian population [49]. Rizk et al. reported that from 114 Arab pregnant women and 45 non-Arab pregnant women with GDM, *TCF7L2 rs 7903146* polymorphism showed no association with GDM but the T allele of *rs 12255372* showed a significant association with GDM in comparison to healthy controls [50].

## Conclusion

In conclusion, we detected a significantly high prevalence of CT/TT genotypes in *rs7903146* and GT/TT genotypes in *rs12255372* of *TCF7L2* gene in women with GDM compared to healthy pregnant women. A good overview of genetic influences might assist in finding a clear path for early detection and efficient management plans regarding GDM. The results from this study will probably help to highlight the possible roles of those variants in our population in developing GDM; yet, wider base studies are needed to find other possible risk factors that increase the prevalence of GDM in the Egyptian population.

## Data Availability

The datasets generated and/or analyzed during the current study are not publicly available due to patient’s privacy but are available from the corresponding author on reasonable request.

## Abbreviations

BMI: Body mass index
GDM: Gestational diabetes mellitus
GLP-1: Glucagon-like peptide-1
HbA1c: Glycated hemoglobin
HDL: High-density lipoprotein
HW: Hardy–Weinberg
IADPSG: International Association of Diabetes and Pregnancy Study Groups
LDL: Low-density lipoprotein
OGTT: Oral glucose tolerance test
SNPs: Single nucleotide polymorphisms
TCh: Total cholesterol
TCF7L2: Transcription factor 7-like 2
TG: Triglycerides
T2D: Type 2 diabetes

## References

[1] ADA (2013). Diagnosis and classification of diabetes mellitus.

[2] Lende M, Rijhsinghani A (2020) Gestational diabetes: overview with emphasis on medical management. Int J Environ Res Public Health 17. 10.3390/ijerph17249573 10.3390/ijerph17249573 PMC7767324 33371325

[3] Hartling L, Dryden DM, Guthrie A, Muise M, Vandermeer B, Aktary WM et al (2012) Screening and diagnosing gestational diabetes mellitus. Evid Rep Technol Assess(210):1–327 (Full Rep) PMC4781607 24423035

[4] Moyer VA (2014) Screening for gestational diabetes mellitus: U.S. Preventive Services Task Force recommendation statement. Ann Intern Med. 10.7326/m13-2905 10.7326/M13-2905 24424622

[5] Barakat MN, Youssef RM, Al-Lawati JA (2010) Pregnancy outcomes of diabetic women: charting Oman’s progress towards the goals of the Saint Vincent Declaration. Ann Saudi Med. 10.4103/0256-4947.65253 10.4103/0256-4947.65253 PMC2931776 20622342

[6] Khalil NA (2017) Screening for gestational diabetes among pregnant women attending a rural family health center- Menoufia Governorate- Egypt. J Fam Med Heal Care. 10.11648/j.jfmhc.20170301.12

[7] Guariguata L, Whiting DR, Hambleton I, Beagley J, Linnenkamp U, Shaw JE (2014) Global estimates of diabetes prevalence for 2013 and projections for 2035. Diabetes Res Clin Pract. 10.1016/j.diabres.2013.11.002 10.1016/j.diabres.2013.11.002 24630390

[8] Lende M, Rijhsinghani A. Gestational Diabetes: Overview with Emphasis on Medical Management. Int J Environ Res Public Health. 2020;17(24):9573. 10.3390/ijerph17249573. 10.3390/ijerph17249573 PMC7767324 33371325

[9] Metzger BE, Gabbe SG, Persson B, Lowe LP, Dyer AR, Oats JJN et al (2010) International Association of Diabetes and Pregnancy Study Groups recommendations on the diagnosis and classification of hyperglycemia in pregnancy: response to Weinert. Diabetes Care. 10.2337/dc09-1848 10.2337/dc09-1848 PMC2827530 20190296

[10] Association AD, ADA (2013). American Diabetes Association: Diagnosis and classification of diabetes mellitus.

[11] Buchanan TA, Xiang A, Kjos SL, Watanabe R (2007) What is gestational diabetes? Diabetes Care. 10.2337/dc07-s201 10.2337/dc07-s201 17596457

[12] Castorino K, Jovanovič L (2011) Pregnancy and diabetes management: advances and controversies. Clin Chem. 10.1373/clinchem.2010.155382 10.1373/clinchem.2010.155382 21148303

[13] Lyssenko V, Lupi R, Marchetti P, Del Guerra S, Orho-Melander M, Almgren P et al (2007) Mechanisms by which common variants in the TCF7L2 gene increase risk of type 2 diabetes. J Clin Invest. 10.1172/JCI30706 10.1172/JCI30706 PMC1934596 17671651

[14] Hansson O, Zhou Y, Renström E, Osmark P (2010) Molecular function of TCF7L2: consequences of TCF7L2 splicing for molecular function and risk for type 2 diabetes. Curr Diab Rep. 10.1007/s11892-010-0149-8 10.1007/s11892-010-0149-8 20878273

[15] Grant SFA, Thorleifsson G, Reynisdottir I, Benediktsson R, Manolescu A, Sainz J et al (2006) Variant of transcription factor 7-like 2 (TCF7L2) gene confers risk of type 2 diabetes. Nat Genet. 10.1038/ng1732 10.1038/ng1732 16415884

[16] Stuebe AM, Wise A, Nguyen T, Herring A, North KE, Siega-Riz AM (2014) Maternal genotype and gestational diabetes. Am J Perinatol. 10.1055/s-0033-1334451 10.1055/s-0033-1334451 PMC3884679 23456907

[17] Boj SF, Van Es JH, Huch M, Li VSW, José A, Hatzis P et al (2012) Diabetes risk gene and Wnt effector Tcf7l2/TCF4 controls hepatic response to perinatal and adult metabolic demand. Cell. 10.1016/j.cell.2012.10.053 10.1016/j.cell.2012.10.053 23260145

[18] Ip W, Shao W, Song Z, Chen Z, Wheeler MB, Jin T (2015) Liver-specific expression of dominant-negative transcription factor 7-like 2 causes progressive impairment in glucose homeostasis. Diabetes. 10.2337/db14-1329 10.2337/db14-1329 25576056

[19] Shaat N, Lernmark Å, Karlsson E, Ivarsson S, Parikh H, Berntorp K et al (2007) A variant in the transcription factor 7-like 2 (TCF7L2) gene is associated with an increased risk of gestational diabetes mellitus. Diabetologia. 10.1007/s00125-007-0623-2 10.1007/s00125-007-0623-2 17342473

[20] Freathy RM, Hayes MG, Urbanek M, Lowe LP, Lee H, Ackerman C et al (2010) Hyperglycemia and Adverse Pregnancy Outcome (HAPO) study: common genetic variants in GCK and TCF7L2 are associated with fasting and postchallenge glucose levels in pregnancy and with the new consensus definition of gestational diabetes mellitus from the I. Diabetes. 10.2337/db10-0177 10.2337/db10-0177 PMC3083839 20682688

[21] Cauchi S, El Achhab Y, Choquet H, Dina C, Krempler F, Weitgasser R et al (2007) TCF7L2 is reproducibly associated with type 2 diabetes in various ethnic groups: a global meta-analysis. J Mol Med. 10.1007/s00109-007-0203-4 10.1007/s00109-007-0203-4 17476472

[22] Lauenborg J, Grarup N, Damm P, Borch-Johnsen K, Jørgensen T, Pedersen O et al (2009) Common type 2 diabetes risk gene variants associate with gestational diabetes. J Clin Endocrinol Metab. 10.1210/jc.2008-1336 10.1210/jc.2008-1336 18984664

[23] Pappa KI, Gazouli M, Economou K, Daskalakis G, Anastasiou E, Anagnou NP et al (2011) Gestational diabetes mellitus shares polymorphisms of genes associated with insulin resistance and type 2 diabetes in the Greek population. Gynecol Endocrinol. 10.3109/09513590.2010.490609 10.3109/09513590.2010.490609 20540670

[24] Cho YM, Kim TH, Lim S, Choi SH, Shin HD, Lee HK et al (2009) Type 2 diabetes-associated genetic variants discovered in the recent genome-wide association studies are related to gestational diabetes mellitus in the Korean population. Diabetologia. 10.1007/s00125-008-1196-4 10.1007/s00125-008-1196-4 19002430

[25] Watanabe RM, Allayee H, Xiang AH, Trigo E, Hartiala J, Lawrence JM et al (2007) Transcription factor 7-like 2 (TCF7L2) is associated with gestational diabetes mellitus and interacts with adiposity to alter insulin secretion in Mexican Americans. Diabetes. 10.2337/db06-1682 10.2337/db06-1682 PMC2925638 17317761

[26] Metzger BE (2010) International Association of Diabetes and Pregnancy Study Groups recommendations on the diagnosis and classification of hyperglycemia in pregnancy. Diabetes Care. 10.2337/dc09-1848 10.2337/dc09-1848 PMC2827530 20190296

[27] Parra-Cordero M, Lees C, Missfelder-Lobos H, Seed P, Harris C (2007) Fetal arterial and venous Doppler pulsatility index and time averaged velocity ranges. Prenat Diagn. 10.1002/pd.1868 10.1002/pd.1868 18000944

[28] Lahiri DK, Numberger JI (1991) A rapid non-enzymatic method for the preparation of HMW DNA from blood for RFLP studies. Nucleic Acids Res. 10.1093/nar/19.19.5444 10.1093/nar/19.19.5444 PMC328920 1681511

[29] Bodhini D, Radha V, Dhar M, Narayani N, Mohan V (2007) The rs12255372(G/T) and rs7903146(C/T) polymorphisms of the TCF7L2 gene are associated with type 2 diabetes mellitus in Asian Indians. Metabolism. 10.1016/j.metabol.2007.04.012 10.1016/j.metabol.2007.04.012 17697858

[30] Szepietowska B, Moczulski D, Wawrusiewicz-Kurylonek N, Grzeszczak W, Gorska M, Szelachowska M (2010) Transcription factor 7-like 2-gene polymorphism is related to fasting C peptide in latent autoimmune diabetes in adults (LADA). Acta Diabetol. 10.1007/s00592-009-0133-4 10.1007/s00592-009-0133-4 19533015

[31] Ye D, Fei Y, Ling Q, Xu W, Zhang Z, Shu J et al (2016) Polymorphisms in TCF7L2 gene are associated with gestational diabetes mellitus in Chinese Han population. Sci Rep. 10.1038/srep30686 10.1038/srep30686 PMC4964615 27465520

[32] Noctor E (2015) Type 2 diabetes after gestational diabetes: the influence of changing diagnostic criteria. World J Diabetes. 10.4239/wjd.v6.i2.234 10.4239/wjd.v6.i2.234 PMC4360417 25789105

[33] Wung SF, Lin PC (2011) Shared genomics of type 2 and gestational diabetes mellitus. Annu Rev Nurs Res. 10.1891/0739-6686.29.227 10.1891/0739-6686.29.227 22891507

[34] Reyes-López R, Pérez-Luque E, Malacara JM. Metabolic, hormonal characteristics and genetic variants of TCF7L2 associated with development of gestational diabetes mellitus in Mexican women. Diabetes Metab Res Rev. 2014;30(8):701-6. 10.1002/dmrr.2538. 10.1002/dmrr.2538 24639413

[35] Huerta-Chagoya A, Vázquez-Cárdenas P, Moreno-Macías H, Tapia-Maruri L, Rodríguez-Guillén R, López-Vite E et al (2015) Genetic determinants for gestational diabetes mellitus and related metabolic traits in Mexican women. PLoS One. 10.1371/journal.pone.0126408 10.1371/journal.pone.0126408 PMC4431878 25973943

[36] Wang B, Xue X (2020) Investigations of associations between seven gene polymorphisms and gestational diabetes mellitus: evidence from a meta-analysis. Gynecol Obstet Investig. 10.1159/000505453 10.1159/000505453 32248196

[37] Guinan KJ (2012) Worldwide distribution of type II diabetes-associated TCF7L2 SNPs: evidence for stratification in Europe. Biochem Genet. 10.1007/s10528-011-9456-2 10.1007/s10528-011-9456-2 21898192

[38] Bryson CL, Ioannou GN, Rulyak SJ, Critchlow C (2003) Association between gestational diabetes and pregnancy-induced hypertension. Am J Epidemiol. 10.1093/aje/kwg273 10.1093/aje/kwg273 14652299

[39] Vysočanová M, Floriánová A, Špinar J (2018) Hypertension in pregnancy. Kardiol Rev. 10.7175/cmi.v1i1.615

[40] Rezaei M, Palizban A, Zamani-Doabi S, Shojaee M (2016) Transcription factor 7-like 2 (TCF7L2) gene polymorphism rs7903146 is associated with lipid profile and risk of cardiovascular disease in metabolic syndrome subjects. J Biol Todays World. 10.15412/J.JBTW.01050703

[41] Potasso L, Perakakis N, Lamprinou A, Polyzou E, Kassanos D, Peter A et al (2020) Clinical impact of the TCF7L2 gene rs7903146 type 2 diabetes mellitus risk polymorphism in women with gestational diabetes mellitus: impaired glycemic control and increased need of insulin therapy. Exp Clin Endocrinol Diabetes. 10.1055/a-1008-9223 10.1055/a-1008-9223 31546272

[42] Reyes-López R, Perez-Luque E, Malacara JM (2019) Relationship of lactation, BMI, and rs12255372 TCF7L2 polymorphism on the conversion to type 2 diabetes mellitus in women with previous gestational diabetes. Gynecol Endocrinol. 10.1080/09513590.2018.1531984 10.1080/09513590.2018.1531984 30614312

[43] Reyes-López R, Pérez-Luque E, Malacara JM (2014) Metabolic, hormonal characteristics and genetic variants of TCF7L2 associated with development of gestational diabetes mellitus in Mexican women. Diabetes Metab Res Rev. 10.1002/dmrr.2538 10.1002/dmrr.2538 24639413

[44] Chang S, Wang Z, Wu L, Lu X, Shangguan S, Xin Y et al (2017) Association between TCF7L2 polymorphisms and gestational diabetes mellitus: a meta-analysis. J Diabetes Investig. 10.1111/jdi.12612 10.1111/jdi.12612 PMC5497039 28002648

[45] Lin PC, Lin WT, Yeh YH, Wung SF (2016) Transcription factor 7-like 2 (TCF7L2) rs7903146 polymorphism as a risk factor for gestational diabetes mellitus: a meta-analysis. PLoS One. 10.1371/journal.pone.0153044 10.1371/journal.pone.0153044 PMC4825985 27058589

[46] Francaite-Daugeliene M, Lesauskaite V, Tamosiunas A, Jasukaitiene A, Velickienė D (2021) Genetic variants of TCF7L2 gene and its coherence with metabolic parameters in Lithuanian (Kaunas district) women population with previously diagnosed gestational diabetes mellitus compared to general population. Diabetes Res Clin Pract 172. 10.1016/j.diabres.2020.108636 10.1016/j.diabres.2020.108636 33352264

[47] de Melo SF, Frigeri HR, dos Santos-Weiss ICR, Réa RR, de Souza EM, Alberton D et al (2015) Polymorphisms in FTO and TCF7L2 genes of Euro-Brazilian women with gestational diabetes. Clin Biochem. 10.1016/j.clinbiochem.2015.06.013 10.1016/j.clinbiochem.2015.06.013 26102344

[48] Gorczyca-Siudak D, Michalak-Wojnowska M, Gorczyca T, Mosiewicz B, Kwaśniewska A, Filip A et al (2016) Association between rs7901695 and rs7903146 polymorphisms of the TCF7L2 gene and gestational diabetes in the population of Southern Poland. Ginekol Pol. 10.5603/GP.2016.0081 10.5603/GP.2016.0081 27958632

[49] Thomas N, Mahesh DM, Chapla A, Paul J, Shwetha N, Christina F et al (2014) Does TCF7L2 polymorphisms increase the risk of gestational diabetes mellitus in South Indian population? Endocr Abstr. 10.1530/endoabs.34.p270

[50] Rizk N (2011) The Associations of Transcription Factor 7-like 2 [TCF7L2] Gene with gestational diabetes mellitus in State of Qatar. Qatar Found Annu Res Forum Proc. 10.5339/qfarf.2011.bmp8

